# Supplementary material for: Association between oral microbiome and seven types of cancers in East Asian population: a two-sample Mendelian randomization analysis
Source: Front Mol Biosci. 2023 Nov 21;10:1327893. doi: 10.3389/fmolb.2023.1327893 (PMC10702768; doi:10.3389/fmolb.2023.1327893)
Supplement: Supplementary file 1 [file DataSheet1.ZIP › Supplementary Materials/MR plots of saliva/saliva═╝/Breast cancer/pheno.1561_to_breast cancer_forest.pdf]

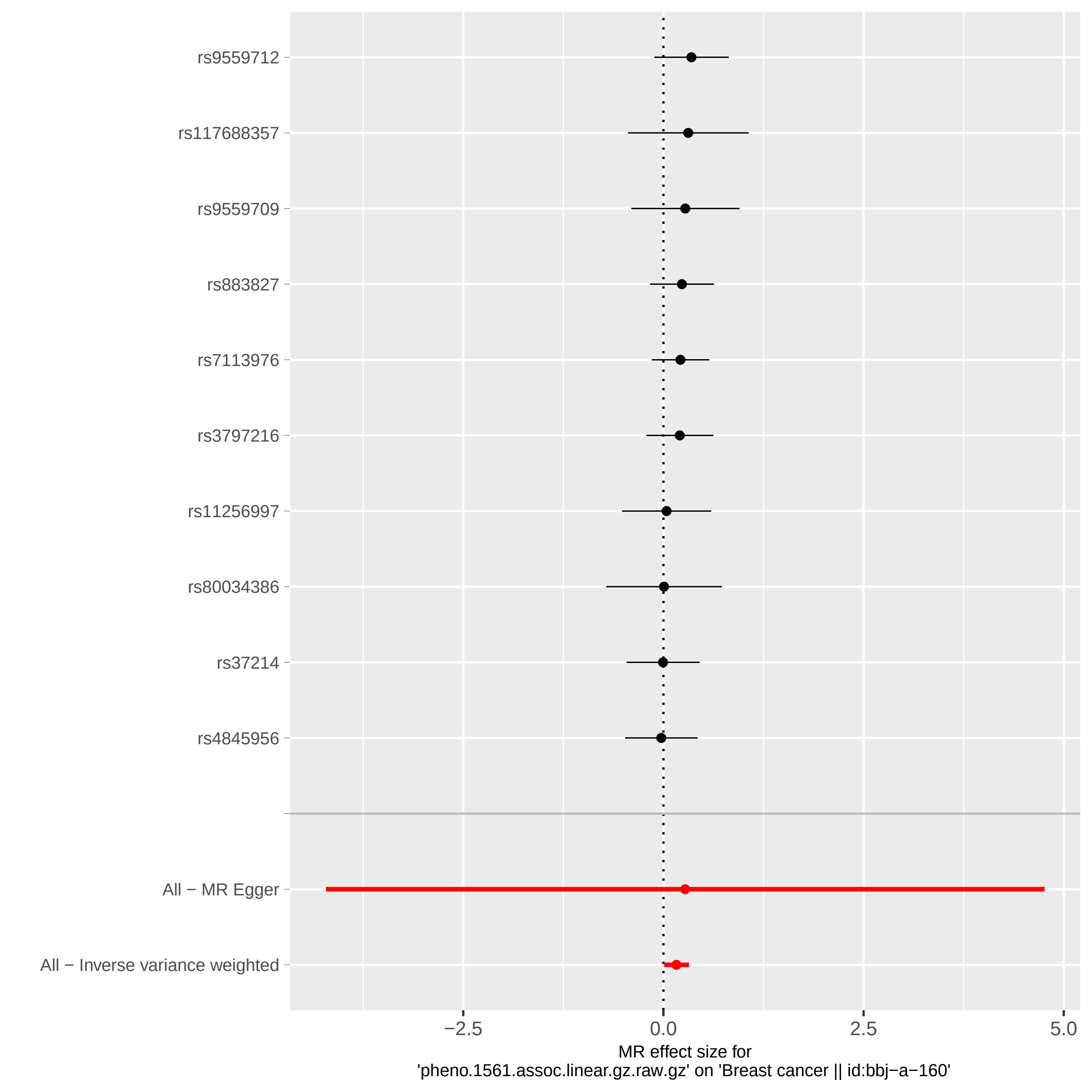
rs9559712
rs117688357
rs9559709
rs883827
rs7113976
rs3797216
rs11256997
rs80034386
rs37214
rs4845956
All − MR Egger
All − Inverse variance weighted
−2.5
0.0
2.5
5.0
MR effect size for
'pheno.1561.assoc.linear.gz.raw.gz' on 'Breast cancer || id:bbj−a−160'
